# Supplementary material for: Enhancing polytetrafluoroethylene (PTFE) coated film for food processing: Unveiling surface transformations through oxygenated plasma treatment and parameter optimization using response surface methodology
Source: PLoS One. 2024 May 31;19(5):e0303931. doi: 10.1371/journal.pone.0303931 (PMC11142506; doi:10.1371/journal.pone.0303931)
Supplement: Attachment — Submitted filename: Rev comments_PONE-D-24-05867.docx [file pone.0303931.s001.docx]

**Review Report**

for the Manuscript entitled: “Enhancing Polytetrafluoroethylene (PTFE) Coated Film for Food Processing: Unveiling Surface Transformations through Oxygenated Plasma Treatment and Parameter Optimization using Response Surface Methodology”

The paper focuses on a topical issue to reduce difficulties due to inherent stickiness arising due to the existence of sugars and organic acids with low molecular weight and a low glass transition temperature. This study expands the knowledge base on the application of oxygen plasma treatment on PTFE coated substrates to improve the efficiency of the spray drying process in order to provide a cost-effective solution for the food industry.

The purpose of the research is clearly stated, the objectives are well established and ambitious, the literature review has been critically followed in line with the state of the art.

### The study is well-designed and well-written, the methods are suitable and the obtained results are well aligned with the purpose of this work. The research methodology is adequate, well described and sufficiently detailed.

The results are well illustrated in 7 Figures and 8 Tables. Statistical data processing is well performed.

The results are clearly presented. Discussions generally provide relevant explanations for obtained results. Some improvements need to be made in the discussions to better highlight the added value of the research.

Conclusions are drawn accordingly based on the data presented and highlight well the results of this study.

The references are relevant to this research topic.

Some aspect can be improved:

The innovation of this study is not well highlighted.

A more description is needed to better reveal the added value of this work.

What are the advantages, disadvantages and limitations of this approach?

The study has high application potential, but also contributes significantly to the development of the knowledge base in the field. Since this approach is of high quality and has great potential for both science and applications, I recommend its publication after a minor review.
