# Supplementary material for: Enhancing polytetrafluoroethylene (PTFE) coated film for food processing: Unveiling surface transformations through oxygenated plasma treatment and parameter optimization using response surface methodology
Source: PLoS One. 2024 May 31;19(5):e0303931. doi: 10.1371/journal.pone.0303931 (PMC11142506; doi:10.1371/journal.pone.0303931)
Supplement: Attachment — Submitted filename: Response to Reviewers 26 April 2024.docx [file pone.0303931.s002.docx]

Dear Sara Hemati
Academic Editor
PLOS ONE

Thank you for the prompt review of our manuscript (Ref. No.: PONE-D-24-05867) entitled **Enhancing Polytetrafluoroethylene (PTFE) Coated Film for Food Processing: Unveiling Surface Transformations through Oxygenated Plasma Treatment and Parameter Optimization using Response Surface Methodology**. We are obliged to revise the manuscript as required and to resubmit it for publication in your **PLOS ONE.** We acted accordingly to improve the manuscript based on the comments of reviewers and resubmit the corrected manuscript as attached. Attached herewith are the lists of corrections for your kind perusal.

**REVIEWER #1**

Changes in the manuscript are highlighted in GREEN colour

| **Reviewer’s Comments and Questions** | **Correction and Answer** |
| --- | --- |
| Abstract:  The excess description in the last three sentences of the abstract needs to be minimized and should be more conclusion-oriented. | 1. The sentences have been mimimized as requested. 2. Changes were carried out accordingly as highlighted in GREEN colour. |
| Introduction:  The introduction looks like a literature review. Could you please compare the areas of advancement of your current studies with the relevant articles cited in each paragraph? | 1. Thank you for your kind comment. 2. Changes were carried out in the Introduction section accordingly as highlighted in GREEN colour. |
| Materials and Methods:  Well written. | Thank you for the positive comments. |
| Result and Discussion:  Well written. | Thank you for your kind attention and positive comments. |
| Conclusion:  Improve the language and quality of the keynotes in this section for the readers. Rewrite the closing statements. | Many thanks for the positive comments. We have rephrased the manuscript, corrected the sentences and its grammatical order to the best of our knowledge and ability. |

**REVIEWER #2**

Changes in the manuscript are highlighted in YELLOW colour

| **Reviewer’s Comments and Questions** | **Correction and Answer** |
| --- | --- |
| The purpose of the research is clearly stated, the objectives are well-established and ambitious, the literature review has been critically followed in line with the state of the art. | Many thanks for the positive comment. |
| The study is well-designed and well-written, the methods are suitable and the obtained results are well aligned with the purpose of this work. The research methodology is adequate, well-described and sufficiently detailed. | A massive thank you for all of your help and passion for reviewing this manuscript. |
| The results are well illustrated in 7 Figures and 8 Tables. Statistical data processing is well performed. The results are clearly presented. | Thank you for your attention and positive comments. |
| Discussions generally provide relevant explanations for obtained results.   - Some improvements need to be made in the discussions to highlight the added value of the research better | We have revised the discussion section and inserted a few passages as follows:  (Line 479)  ‘Furthermore, desorption isotherms can be acquired through either graphical or numerical integration methods (Naderi, 2015). In materials science and chemistry, BET surface area analysis is a crucial technique for assessing the surface properties of various materials.’  (Line 457)  ‘Based on the above mentioned finding, the Raman spectroscopy serves as a valuable tool for authenticating food products by identifying distinctive molecular fingerprints linked to specific ingredients or characteristics. Food manufacturers can maintain product quality and safety by monitoring these surface alterations and adjusting processing and storage conditions accordingly.’  (Line 325)  ‘Water Contact Angle (WCA) underscores the significance of precise measurement and control of surface properties to optimize performance and facilitate innovative applications, including mechanisms for altering surface hydrophobicity.’ |
| Conclusions are drawn accordingly based on the data presented and highlight well the results of this study.  The references are relevant to this research topic. | Many thanks for the positive comment. |
| The innovation of this study is not well highlighted.   - More description is needed to better reveal the added value of this work. | We revised this manuscript and added a few sentences as requested.  (Line 544)  ‘This finding holds relevance for industries like food processing and pharmaceuticals, emphasizing the importance of controlling the drying process for superior product quality.  (Line 528)  ‘Plasma treatment, particularly with oxygen plasma, can make originally hydrophobic (water-repelling) surfaces more hydrophilic (water-loving). This is beneficial for applications like spray drying, where powder need to be spread and collected properly to the maximum yield’.  (Line 547)  ‘In contrast to conventional solvent-based surface allteration techniques, plasma treatment offers a more environmentally sustainable alternative. It produces minimal waste and operates through a dry process. |
| What are the advantages, disadvantages, and limitations of this approach? | Thank you for your suggestion. The addition of phrases was made accordingly based on line distribution.  (Line 131)  ‘Although plasma treatment presents numerous benefits for surface modification, it also comes with certain limitations and potential drawbacks. Plasma operates by subjecting the surface to high-energy particles. Excessive power or prolonged treatment durations can result in surface damage, causing etching, cracking, or weakening of the material. Moreover, there is a risk of uneven treatment, with some areas being over-treated while others receive insufficient modification.’  (Line 556)  ‘Surface modification via plasma treatment offers numerous benefits, making it a valuable asset across various industries. These advantages enhance product performance and functionality significantly.’ |

Best regards,

**Assoc. Prof. Ts. Dr. SAIFUL IRWAN ZUBAIRI**

Ph.D (Chemical Engineering) Imperial College London, M.Eng. (Bioprocess), B.Eng. (Hons.)(Chemical-Bioprocess) UTMalaysia | P.Tech. (MBOT), AMIChemE, PMIFT, Grad. (BEM)

Department of Food Sciences, Faculty of Science & Technology,

Universiti Kebangsaan Malaysia, 43600 UKM Bangi, Selangor.

+603-8921-5989 | [saiful.zubairi08@alumni.imperial.ac.uk](mailto:saiful.zubairi08@alumni.imperial.ac.uk) | [saiful-z@ukm.edu.my](mailto:saiful-z@ukm.edu.my)
